# Sensory organization during balance and gait in people with and without chronic ankle instability

**DOI:** 10.3389/fspor.2025.1569407

**Published:** 2025-07-02

**Authors:** W. J. Decker, J. Henrickson, M. Mukherjee, C. J. Burcal, A. B. Rosen

**Affiliations:** ^1^ School of Health and Kinesiology, University of Nebraska at Omaha, Omaha, NE, United States; ^2^ Department of Physical Therapy, University of Nebraska Medical Center, Omaha, NE, United States; ^3^ Department of Biomechanics, University of Nebraska at Omaha, Omaha, NE, United States

**Keywords:** sensory reweighting, visual reliance, gait, spatiotemporal variability, balance (static)

## Abstract

Lateral ankle sprains are the most common musculoskeletal injury and can develop into chronic ankle instability (CAI). People with CAI rely more on visual and somatosensory information to maintain stability in static and dynamic tasks. Researchers use the Sensory Organization Test (SOT) to systematically perturb the visual and somatosensory inputs to assess sensory reweighting through changes in double-leg balance in six increasingly difficult conditions. Similarly, the Locomotor Sensory Organization Test (LSOT) perturbs visual and somatosensory inputs to assess sensory reweighting during gait in six increasingly difficult conditions. The purpose of this study was to determine if there was a difference in SOT and LSOT performance in individuals with CAI compared to uninjured, healthy controls. Forty-four individuals with (*n* = 23) and without (*n* = 21) self-reported CAI were assessed in both the SOT and the LSOT. The primary outcome of SOT performance was measured using an equilibrium score that evaluates the movement of center of pressure. The primary outcomes of LSOT performance were assessed by calculating spatiotemporal gait variables including cycle, stance, and swing time and stride length and width. Separate 2 × 6 generalized linear mixed model ANOVAs were performed for the SOT and LSOT to examine the main effects of Condition, Group, and their interaction. Both the SOT and the LSOT showed a significant Condition main effect, indicating altered motor output as sensory systems were progressively perturbed. Additionally, the SOT showed a significant Group × Condition interaction, indicating that the CAI group showed better balance in condition 5 than the uninjured, healthy control group. These findings suggest that both somatosensory and visual perturbations influence balance equally in both groups, as no between-group differences were observed on the SOT. During the LSOT we observed a Condition main effect with significant differences in the spatiotemporal variables across conditions, with treadmill belt speed perturbations causing the largest disruption to gait outcomes. There were no differences between groups, indicating that both people with and without CAI choose stability when possible to properly navigate perturbations during gait. This data suggests that progressive sensory perturbations alter movement in individuals with and without CAI during constrained tasks.

## Introduction

Lateral ankle sprains (LAS) are one of the most common musculoskeletal injuries among individuals who participate in sport or recreational activity (1). The resulting pain and dysfunction of the ankle joint can develop into chronic ankle instability (CAI) (2), and research has shown that up to 70% of LAS develop CAI (3). CAI is characterized by functional impairments (4) such as ongoing pain, weakness, decreased range of motion (ROM), and diminished self-perceived function of the ankle. It is also characterized by instability due to repetitive episodes of giving way (5) after initial injury or recurrent ankle sprains (6). Delahunt et al. (5) defines an episode of giving way as an unexplained instance of ankle inversion that does not result in a LAS. CAI is often accompanied by sensorimotor dysfunction, characterized by balance (7) and gait alterations (8) that may contribute to reinjury such as an increased reliance on the visual system (9, 10) to help maintain stability during static balance and gait, and an increased inversion angle due to ligament laxity in the lateral ankle. It is thought that these sensorimotor impairments are the result of central nervous system (CNS) adaptations also defined as maladaptive neuroplasticity. Needle et al. (11) argue that maladaptive neuroplastic changes can result in altered motor planning during certain activities, leaving an individual vulnerable to reinjury (12). The researchers go on to theorize that these motor adaptations could contribute to long-term joint degeneration.

One theory as to why CAI symptoms persist even after rehabilitation is that maladaptive changes as a result of persistent reinjury lead to altered sensory reweighting (10) in the CNS (12). As CAI develops, there is an increased use of and reliance on the visual system (10) for cues to guide movement as the somatosensory system (13, 14) becomes dysfunctional. Injury to the ankle joint and surrounding peripheral nerves may explain poor proprioception (12), joint position sense, and cutaneous sensitivity (13, 15) at the foot and ankle. This could place an increased burden on the visual and vestibular systems to help maintain stability (16). The increased latency required to process visual cues can limit the rate at which the body can detect perturbations and make postural corrections or tend to other tasks (e.g., sports), leading to a sense of instability (17). Such a shift in sensory prioritization could contribute to altered biomechanics of walking (11, 12) and running in people with CAI (18). To better understand the extent of these adaptations and the role of sensory input in movement, studies should use methods to perturb each sensory modality systematically.

The most common method used in the literature to evaluate sensory reweighting is comparing eyes-open to eyes-closed conditions while maintaining balance, however, this is limited as it only perturbs one sensory modality—vision (10). One method to holistically assess sensory reweighting is the Sensory Organization Test (SOT). The SOT (19) is a technique that researchers use to systematically perturb the visual and somatosensory inputs to assess sensory reweighting (20) through changes in balance performance across six increasingly difficult conditions by providing an equilibrium score to represent balance performance. Research has shown (19, 20) that as the sensory systems are increasingly perturbed, balance worsens. A drawback to the SOT is that it assesses sensory reweighting during standing double-leg balance, a relatively stable motor task. To examine these postural control strategies during a more dynamic movement such as walking, the Locomotor Sensory Organization Test (LSOT) (21) was developed. With similar conditions as the SOT, the LSOT uses both a split treadmill and an immersive virtual reality screen to alter the visual and somatosensory inputs during gait. Although researchers have performed nonlinear analysis on the LSOT (21), other gait spatiotemporal variables can be assessed, such as total cycle time, stance time, swing time, stride length, and stride width. These spatiotemporal gait outcomes may provide insight into the strategies used during gait when adapting to sensory and/or perceptual constraints. For example, to increase stability, an individual could alter their gait to increase stance time, stride width and decrease stride length and swing time when faced with uncertain sensory conditions (18). Previous research (22) has found evidence that people with CAI display a more constrained pattern of gait initiation, suggesting motor planning strategies that optimize stability. While prior work has studied the effects of the SOT on postural stability during standing balance (19, 20), little is known about how those with CAI adapt gait to sensory and/or perceptual constraints. The LSOT could generate useful insight as most of the recurrent injury episodes in people with CAI occur during dynamic movements and it remains to be seen how adaptive sensory reweighting alters motor performance during a non-static task.

Therefore, the purpose of this study was to determine if there was a difference in performance during the SOT and LSOT in individuals with CAI and uninjured controls. We hypothesized that those with CAI would have a smaller equilibrium score in each condition compared to the uninjured, healthy control participants, Additionally, we hypothesized that those with CAI would have a more drastic decrease in equilibrium scores as sensory systems became more perturbed with each subsequent condition compared to the uninjured, healthy control participants. For the LSOT, we hypothesized that those with CAI would have a longer cycle time, a longer stance time, a shorter stride length, a wider stride width, and a shorter swing time than uninjured, healthy controls. We believed these changes to gait would occur as those with CAI attempted to employ strategies to have a more stable gait during conditions of sensory uncertainty.

## Methods

A total of 44 physically active young adults were recruited to take part in this study and were split into two different groups: CAI (*n* = 23) and an uninjured, healthy control group (*n* = 21) (Table 1). Prior to testing, each participant signed an informed consent approved by the University's Institutional Review Board. Both the inclusion and exclusion criteria for each group were based on the position statement of the International Ankle Consortium (23). To be included in the CAI group, participants must have met the following criteria: (1) history of at least one significant ankle sprain(s) that required at least three days of partial- or non-weight bearing activity; (2) a history of at least two episodes of “giving way”, and a general feeling of instability in the ankle joint; and (3) a score of ≤24 on the Cumberland Ankle Instability Tool (CAIT) (24). To be included in the uninjured, healthy control group, the individuals must: (1) have no history of ankle sprains, and (2) a score of 30 on the CAIT, indicating no ankle dysfunction. Individuals were excluded from the study if they met any of the following: (1) evidence of current injury (pain, heat, redness, discoloration) of the lower extremity; (2) previous history of lower extremity surgery; (3) previous history of broken/fractured bones in the lower extremity; (4) a diagnosis of a vestibular disorder or connective tissue disorder; (5) currently taking medications that affect cognitive function or balance; and 6) not participating in a minimum of 90 min of physical activity per week.

**Table 1:** Subject demographics.

	Groups		*P*
	CON	CAI	
*N*	21 (9 F, 12 M)	23 (12 F, 12 M)	—
Age (years)	21.8 (2.2)	22.2 (2.2)	0.546
Height (cm)	173.9 (8.)	174.0 (8.7)	0.966
Mass (kg)	77.9 (13.2)	74.1 (14.1)	0.370
BMI (kg/m^2^)	25.6 (2.7)	24.3 (3.5)	0.181
CAIT	30 (0.0)	18.2 (3.7)	<0.001*
# of Sprains	0.00 (0.00)	4.35 (4.3)	<0.001*
PWS (m/s)	1.16 (.05)	1.15 (.04)	0.693

Mean (SD).

CON, control group; CAI, chronic ankle instability; BMI, body mass index; F, female; M, male; CAIT, Cumberland Ankle Instability Tool; PWS, preferred walking speed.

#of Sprains is the total # of sprains each participant has experienced.

* Denotes statistically significant (*p* *<* 0.05) difference between groups.

### Sample size justification

A power analysis utilizing data from internal pilot testing was completed with G*Power (Version 3.0.10 Kiel University, Germany) to determine the appropriate sample size necessary to detect significant differences among the dependent variables of interest. A between-groups effect size of 0.63, combined with an *α* = .05 and 1 − *β* = .80 an a-priori power calculation indicated 36 total participants were necessary to determine significant differences.

### Protocol

After providing informed consent, participants began the test protocol across 2 sessions. Participants first completed the SOT and then they returned to the laboratory to complete the LSOT a week later.

### Sensory organization test

The SOT used the *Balance Master System 8.4* (NeuroCom International Clackamas, OR, USA). This system contains a movable visual surround and support surface that rotates in the anterior-posterior (AP) plane. Two force plates are used to collect CoP data at 100 Hz. While using the Balance Master system, subjects wore a safety harness to prevent falling, which did not interfere with the participants’ natural postural sway and allowed for CoP data collection.

The SOT protocol consists of 6 different conditions performed in order (25). Condition 1 uses a fixed platform and surround and is the only condition that assesses the contributions of all three sensory modalities—vision, somatosensory, and vestibular. Condition 2 uses a fixed platform with the eyes closed and assesses the involvement of the somatosensory and vestibular systems, condition 3 uses a fixed platform but perturbs the visual system by moving the surround with respect to sway. Condition 4 perturbs the somatosensory system by moving the platform with respect to sway, condition 5 assesses the contributions of a perturbed somatosensory system and the vestibular system by moving the platform with respect to sway with the eyes closed, and condition 6 perturbs the visual and somatosensory systems by moving the surround and platform with respect to sway. Each condition consisted of 3 trials of 20 s. The NeuroCom system calculates an equilibrium score for the anterior-posterior direction by assessing how well the participant maintains their position within the theoretical limits of stability in that plane. A composite equilibrium score (20) is then calculated by using a weighted average of all conditions with more difficult conditions (3–6) receiving higher weights (26). A higher composite equilibrium score indicates a higher level of postural stability. Scores range from 100 to 0 with 100 indicating perfect balance and 0 indicating a lack of balance. The 3-trial average for each condition was used in statistical analysis.

### Locomotor sensory organization test

The LSOT was performed using a Gait Real-time Analysis Interactive Lab (GRAIL) (Motek, Amsterdam, Netherlands), which consisted of three components: 16 cameras for 3D motion tracking, a high-definition video (Vicon, Oxford, UK), and an instrumented treadmill (Bertec Corp., Columbus, OH, USA). The treadmill was a split-belt design with tracks for the left and right legs. It included a 180-degree screen and front-mounted projectors for complete visual immersion. The treadmill contained embedded force plates (1000 Hz) on each belt integrated into a single system to allow for synchronized collection of kinetic data. Similar to the SOT, the LSOT consists of six conditions, performed in order (25), to manipulate sensory information while walking: condition 1 is normal walking, condition 2 reduces the visual field by reducing vision capability through the use of light intensity goggles (MSA Safety Work, Pittsburgh, PA, USA), and condition 3 perturbs the visual field by manipulating optic flow speed between 80% and 120% of preferred walking speed (PWS). Condition 4 perturbs the somatosensory system by manipulating treadmill speed between 80% and 120% PWS, condition 5 perturbs the visual and somatosensory systems by reducing vision capability and manipulating treadmill speed between 80% and 120% PWS, and condition 6 perturbs the visual and somatosensory systems by manipulating optic flow and treadmill speed between 80% and 120% PWS (21).

During the LSOT, subjects wore a tight-fitting spandex singlet with a lower body Plug-In Gait marker set using 16 retroreflective markers that was placed on their lower extremities (left/right anterosuperior iliac spine, left/right posterosuperior iliac spine, lateral thigh, knee, shank, ankle toe, and heel) (27, 28). Three-dimensional marker positions were captured from the retroreflective markers at 100 Hz using an 16-camera motion capture system (Vicon, Oxford, UK). Motion capture data was recorded using Vicon Nexus software version 2.2.5 (Vicon, Oxford, UK).

Subjects stood on the split-belt treadmill (Bertec Corp., Columbus, OH, USA) and were secured into the safety harness (Solo-Step, Inc., North Sioux City, SD, USA). The safety harness did not hinder nor assist the subject's gait. They were instructed to maintain eye contact with the virtual environment after being placed on the treadmill to avoid desensitization to the virtual reality environment. The participants PWS was determined by slowly increasing the walking speed until a comfortable speed was found during a 5-min adaption period to acclimate to the virtual environment and walking on the split-belt treadmill (29). After the adaptation period, subjects walked for each of the six LSOT conditions. Each condition was 125 s long and were allowed a minimum 1-min rest in between conditions.

### Data processing

For the LSOT trial, the first five seconds were removed to ensure a normal gait pattern after starting each condition (30); this was done to remove the acute adaptations to each new condition. The unfiltered position data for the *x*, *y*, and *z* coordinates were exported and lowpass filtered with a cutoff frequency of 6 Hz (31). Spatiotemporal gait parameters were calculated using Visual 3D (HAS-Motion, Germantown, MD, USA). Gait events were determined using a ground reaction force threshold of 10 N (29). Successive unilateral and bilateral events—left and right toe off, left and right heel strike—were used to calculate the spatiotemporal parameters. The spatiotemporal outcomes calculated included gait cycle time, stance time, swing time, stride length, and stride width.

### Statistical analysis

Separate 2 × 6 Mixed Model ANOVAs were performed using jamovi (Version 2.3.28.0), where the between-subjects factor was the Group (CAI vs Uninjured Control) and the within-subjects factor was the Condition (1–6) of both the SOT and LSOT. The dependent variable for the SOT was the equilibrium score and for the LSOT we analyzed spatiotemporal parameters (gait cycle time, stance time, swing time, stride length, stride width). The Shapiro-Wilk test was performed to determine normality and Mauchly's Test of Sphericity to determine homogeneity of each sample (*p* < 0.05); these tests indicated data were not normally distributed, thus violating the assumptions of a Mixed Model ANOVA. Thus, to evaluate the effects of sensory reweighting for the SOT and the five outcomes from the LSOT, the distribution of the data during analysis was changed from Gaussian to Gamma distribution to counteract the effect of non-normality (32), where a generalized linear mixed model ANOVA was performed. A Bonferroni *post-hoc* correction was applied to determine where the differences between groups were. Alpha was set at 0.05.

## Results

Statistical analysis of the SOT (Table 2) revealed a significant Condition main effect (chi-squared = 989.70, *p* < 0.001) and a significant Condition × Group interaction effect (chi-squared = 18.70, *p* = 0.002). There was no significant Group main effect (chi-squared = 0.00134, *p* = 0.971). The Condition main effect revealed significant differences between conditions 1 and 4 (*p* < 0.001), 5 (*p* < 0.001), and 6 (*p* < 0.001), conditions 2 and 4 (*p* < 0.001), 5 (*p* < 0.001), and 6 (*p* < 0.001), conditions 3 and 4 (*p* < 0.001), 5 (*p* < 0.001), and 6 (*p* < 0.001), and conditions 4 and 5 (*p* < 0.001), and 6 (*p* < 0.001). Upon evaluation of the *post-hoc* values of the interaction effects, the only significant difference in the equilibrium scores found was between condition 1 and condition 5 (*p* = 0.05). Interestingly, in condition 5, those within the CAI group had a higher mean equilibrium score (62.9) than those in the uninjured control group (57.5).

**Table 2:** Equilibrium scores across conditions of SOT.

Condition	CON Mean (SD) (*n* = 21)	CAI Mean (SD) (*n* = 23)
1	94.98 (1.45)	95.06 (1.83)
2	92.56 (2.16)	91.75 (3.71)
3	92.49 (2.01)	91.06 (3.84)
4	81.60 (9.91)^b^^,^^c^^,^^d^	77.93 (12.20)^b^^,^^c^^,^^d^
5^a^	58.85 (14.82)^b^^,^^c^^,^^d^^,^^e^	62.65 (8.49)^b^^,^^c^^,^^d^^,^^e^
6	61.56 (15.21)^b^^,^^c^^,^^d^^,^^e^	65.42 (10.93)^b^^,^^c^^,^^d^^,^^e^

Mean (SD).

CON, control group; CAI, chronic ankle instability group.

^a^ Significant interaction effect between control group and those with CAI.

^b^ Significant difference from condition 1.

^c^ Significant difference from condition 2.

^d^ Significant difference from condition 3.

^e^ Significant difference from condition 4.

There were no significant interaction effects during the LSOT (*p* > 0.05). There were, however, significant Condition main effects in cycle time (chi-squared = 55.76, *p* < 0.001) (Table 3), stance time (chi-squared = 24.54, *p* < 0.001) (Table 3), and stride length (chi-squared = 61.95, *p* < 0.001) (Table 4). There was no statistically significant Group main effect (*p* > 0.05). *Post-hoc* analysis of the Condition main effect showed a significant difference between conditions 1 and 3 (*p* = 0.032), 4 (*p* < 0.001), 5 (*p* < 0.001), and 6 (*p* = 0.007), conditions 2 and 4 (*p* < 0.001), and 5 (*p*p = 0.006), conditions 3 and 4 (*p* = 0.007), and conditions 4 and 6 (*p* = 0.032) for cycle time (Table 3). For stance time, analysis showed differences between conditions 1 and 4 (*p* = 0.006), conditions 2 and 4 (*p* < 0.001), conditions 3 and 4 (*p* = 0.032), and conditions 4 and 6 (*p* = 0.002) (Table 3). For stride length, analysis showed differences between conditions 1 and 3 (*p* = 0.011), 4 (*p* < 0.001), 5 (*p* < 0.001), and 6 (*p* = 0.001), conditions 2 and 4 (*p* < 0.001), and 5 (*p* = 0.002), and conditions 3 and 4 (*p* = 0.008) (Table 4). There were no significant differences in swing time (Table 3) or stride width (Table 4) (*p* > 0.05).

**Table 3:** Spatiotemporal parameters across conditions of LSOT.

Condition	Groups	Cycle time (s)	Stance time (s)	Swing time (s)
1	CON	1.09 (0.10)	0.71 (0.07)	0.41 (0.05)
	CAI	1.13 (0.11)	0.74 (0.08)	0.42 (0.06)
2	CON	1.09 (0.17)	0.71 (0.10)	0.49 (0.45)
	CAI	1.11 (0.11)	0.75 (0.10)	0.40 (0.03)
3	CON	1.07 (0.15)^a^	0.71 (0.09)	0.40 (0.10)
	CAI	1.09 (0.10)^a^	0.73 (0.10)	0.39 (0.03)
4	CON	1.03 (0.18)^a^^,^^b^^,^^c^	0.69 (0.11)^a^^,^^b^^,^^c^	0.41 (0.13)
	CAI	1.06 (0.15)^a^^,^^b^^,^^c^	0.71 (0.09)^a^^,^^b^^,^^c^	0.40 (0.05)
5	CON	1.03 (0.15)^a^^,^^b^^,^^d^	0.71 (0.09)	0.39 (0.07)
	CAI	1.07 (0.12)^a^^,^^b^^,^^d^	0.72 (0.09)	0.41 (0.06)
6	CON	1.08 (0.19)^a^	0.73 (0.12)^d^	0.45 (0.27)
	CAI	1.07 (0.11)^a^	0.72 (0.07)^d^	0.41 (0.08)

Mean (SD).

CAI, chronic ankle instability (*n* = 23); CON, control (*n* = 21).

^a^ Significantly different from Condition 1.

^b^ Significantly different from Condition 2.

^c^ Significantly different from Condition 3.

^d^ Significantly different from Condition 4.

**Table 4:** Spatiotemporal parameters across conditions of LSOT.

Condition	Groups	Stride length (m)	Stride width (m)
1	CON	1.27 (0.14)	0.18 (0.04)
	CAI	1.29 (0.13)	0.17 (0.04)
2	CON	1.26 (0.22)	0.19 (0.04)
	CAI	1.27 (0.12)	0.16 (0.03)
3	CON	1.23 (0.20)^a^	0.18 (0.03)
	CAI	1.24 (0.11)^a^	0.16 (0.03)
4	CON	1.18 (0.24)^a^^,^^b^^,^^c^	0.18 (0.03)
	CAI	1.21 (0.16)^a^^,^^b^^,^^c^	0.17 (0.03)
5	CON	1.19 (0.20)^a^^,^^b^	0.19 (0.03)
	CAI	1.23 (0.13)^a^^,^^b^	0.17 (0.03)
6	CON	1.25 (0.25)^a^	0.18 (0.03)
	CAI	1.22 (0.13)^a^	0.17 (0.03)

Mean (SD).

CAI, chronic ankle instability (*n* = 23); CON, control (*n* = 21).

^a^ Significantly different from Condition 1.

^b^ Significantly different from Condition 2.

^c^ Significantly different from Condition 3.

## Discussion

The aim of this study was to determine if there were differences in performance during the SOT and LSOT in individuals with CAI compared to healthy controls. We hypothesized that there would be a difference between groups in each test; this hypothesis was partially supported as we found a significant interaction in the equilibrium scores of the SOT but not the values of the spatiotemporal variables (cycle time, stride length, stride width, stance time, and swing time) of the LSOT. However, within-group analysis showed that both balance and gait were altered in each group (Tables 2–4) as sensory systems were progressively challenged. As expected, balance worsened with increasing sensory challenges, and similar findings were observed in the LSOT as evidenced by the statistically significant main effects of Condition. As inputs to the sensory systems were disrupted via optic flow and speed changes, all participants spent more time in double-leg stance. We found that as sensory systems were increasingly perturbed the total cycle time had a proportional decrease, leading to an increase in double-leg stance time. This effect was most notable during treadmill belt speed perturbations. Such changes could indicate that the participants changed their gait patterns to increase stability while under uncertain or novel sensory conditions. This data supports previous findings (33, 34) that sensory reweighting affects motor outcomes during multiple motor tasks, however this effect seems to be equal in people with and without CAI.

### SOT

The hypothesis of between-group differences in balance on the SOT was only partially supported. The data did not reveal any global differences in SOT performance between groups, however we did identify a significant interaction between Group and Condition indicating that balance during condition 5 was better in people with CAI compared to uninjured, healthy controls. The literature has mixed findings in comparisons of SOT performance between people with and without CAI (33, 34). These results contrast with Song and Wikstrom (33) who reported worse balance in people with CAI on conditions 1, 2, and 5 compared to uninjured, healthy controls. Interestingly, we found that participants with CAI had better balance during condition 5 (no vision, sway-referenced platform movement) than uninjured controls. These results mostly agree with Sugimoto et al. (33), who also reported no differences in SOT performance between groups. These authors argue that CAI participants may have undergone rehabilitation, which may explain nonsignificant differences between groups. While this could have affected balance, the present study and those referenced (33, 34) did not require previous rehabilitation as a specific eligibility criterion. Since there was only one comparison within the significant interaction effect, it is unclear if rehabilitation influenced balance in the sample of individuals with CAI, as the results show that balance was not impaired during less drastic sensory perturbations.

Another possible explanation is that the balance task during the SOT is not challenging enough to the sensorimotor system in people with CAI. Double-leg standing balance is a mechanically stable posture with a wide base of support. This may explain why the SOT is more often used to evaluate CNS-based injuries (35, 36) and disorders rather than musculoskeletal conditions. Previous research on sensory perturbations (10) in people with and without CAI show significantly worse balance during single-leg standing, a less stable posture (10). As motor tasks become more difficult, a similar pattern of motor performance trade-offs and task complexity has also been observed during cognitive-motor dual tasking in people with CAI. Recent systematic reviews (18, 37) show that significant between-group differences in motor performance are present during dual-task assessments with more difficult motor or cognitive tasks. While the current data demonstrated balance was better in CAI during a single condition, we feel this data indicates that sensory adaptation during balance occurs similarly between groups. This could suggest that in tasks where there is minimal threat of ankle perturbation, people with CAI appear to reweigh sensory inputs similarly and/or have similar balance trade-offs to uninjured, healthy people. Future studies aiming to leverage the feedback model of balance to assess sensory reweighting should consider using a more challenging stance (e.g., single-leg) to better reveal CAI-specific impairments. Additionally, incorporating a dual-task protocol (37) could help shift the participant's focus away from maintaining balance and toward completing a concurrent task, thereby increasing the sensitivity of the assessment.

### LSOT

Spatiotemporal gait parameters changed throughout each increasingly perturbed condition in each group during the LSOT. This data showed a significant Condition main effect for cycle time, stance time, and stride length (Tables 3, 4). These Condition main effects suggest that timing in the gait cycle was altered due to sensory perturbations. Cycle time shortened as sensory systems were increasingly perturbed, with the most notable changes occurring during condition 4, which altered the treadmill belt speed to force the participants in each group to adapt to walk at different speeds than their PWS. Potentially, cycle time was shortened in response to the changes in belt speed in condition 4. This could reflect different strategies between both people with and without CAI, wherein the gait pattern is altered more by external factors (e.g., treadmill belt speed) rather than internally driven movement patterns. This is further evidenced by the reduction in stance time in condition 4. This is an interesting finding because previous research (18) has shown an increase in stance time in those with CAI as a method to maintain stability during perturbations. Further, it is argued (38) that this increase in stance time is a method those with CAI use to increase the time to respond to any unexpected perturbations. This contrasts with the current study. The decrease in stance time could be an anticipatory strategy aimed at minimizing exposure to potential perturbation by reducing the duration of limb loading, thereby limiting the time available for instability to occur. Another possible explanation is that it could just reflect a change in overall gait cycle time as a result of adapting to the different external cues of each condition. Together these two data points may indicate that people with and without CAI adapt their gait pattern in patterns that may serve to maintain stability when experiencing sensory perturbations.

As the cycle time and stance time decreased across conditions, the stride length also decreased. There were no differences observed between groups, with the Condition main effect meaning that those with and without CAI adapted the same way when walking with different perturbations. These findings support the secondary hypothesis (39, 40). This finding partially contradicts existing CAI literature (39, 40) that found people with CAI have a shorter stride length than people without CAI, whereas we did not observe any between-group differences in any of the gait metrics (Tables 3, 4). However, the Condition main effect indicating decreasing stride length with increasingly difficult sensory perturbations (Table 4) shows that both groups may have altered their gait pattern to prioritize stability. The authors argue that decreasing stride length is a post-injury adaptation in CAI that aims to increase stability; a similar pattern could have occurred here wherein the current observed spatiotemporal changes to gait (Tables 3, 4) served to optimize stability during uncertain sensory conditions.

In the seminal report on the LSOT the researchers performed nonlinear analysis to assess center of mass (CoM) sway patterns (21). Their results show that uninjured young adults adopted a more rigid and predictable sway pattern of the CoM as sensory systems were increasingly perturbed (21). While we cannot directly compare these findings to that of Chien et al. (21), the pattern of increasing rigidity and stability of gait during the LSOT appears to be mostly replicated (Tables 3, 4). A more stable gait pattern may also adopt a wider stride width in response to perturbation (41), yet this data did not reveal any differences in stride width (Table 4). We believe this to be due to the use of a split-belt treadmill, which tends to force people to exhibit a wider base of support (42) compared to walking on a single-belt treadmill or the ground itself. To better evaluate gait adaptation strategies and add descriptive context to how people with CAI adapt to sensory perturbations, future analyses may aim to replicate the CoM trajectory approach of Chien and colleagues (21). Nonlinear analyses enable researchers to compare how much gait deviation occurred with CoM summary outcomes (e.g., mean velocity, displacement) with the structure of how the CoM deviated throughout the sensory perturbations (i.e., regularity of sway using sample entropy). This complementary analysis would allow for better inferences about the postural strategies adopted when people are confronted with sensory perturbations.

### Limitations

The present study was not without limitations. We did not collect a common anchor measure of sensory reweighting as commonly reported in the CAI field (10), a comparison of single-leg eyes-open and eyes-closed balance. By not collecting single-leg balance measures of eyes open and eyes closed balance, we are unable to describe the degree of visual reliance the sample of those with CAI has with respect to known values in the literature (10). Due to the heterogeneity of symptoms and impairments in CAI it is possible that some of the participants in the CAI group did not have an increase in visual reliance. Additionally, it is possible that participants with CAI could have experienced symptoms (e.g., pain, stiffness) between test sessions which could have altered data. Participants were asked if any new symptoms or injuries occurred between sessions, but pain and discomfort were not assessed. Lastly, the motor task of the SOT and LSOT, double-leg standing and split-belt treadmill walking, may have been too constrained. These motor tasks may not be challenging enough to adapt to with varying sensory perturbations, and future research should look to develop and validate assessments that incorporate more challenging (i.e., cognitive loading or single-leg standing) or unconstrained (e.g., above-ground walking or running) motor tasks when investigating sensory organization in physically active populations.

## Conclusions

The current study found that double-leg balance became progressively more unstable in the CAI group and the uninjured, healthy group as the difficulty of the SOT conditions increased, as evidenced by the significant Condition main effect in the SOT. Additionally, we found that as sensory systems become perturbed during the LSOT, an individual's gait becomes much more rigid in an attempt to provide the stability needed to safely complete a task as seen by an increase in stance time, the decrease in cycle time, and the decrease in stride length during the LSOT in all participants across all conditions. These results could indicate that the trade-offs in motor performance are not different between groups and people with CAI adapt to sensory perturbations in a similar manner to people without CAI. Future studies or analyses may want to focus on measures of gait stability to determine if more or less stable movement patterns were utilized during perturbed gait.

## Data Availability

The raw data supporting the conclusions of this article will be made available by the authors, without undue reservation.
